# Supplementary material for: Systematic profiling of temperature- and retinal-sensitive rhodopsin variants by deep mutational scanning
Source: J Biol Chem. 2021 Oct 29;297(6):101359. doi: 10.1016/j.jbc.2021.101359 (PMC8649220; doi:10.1016/j.jbc.2021.101359)
Supplement: Figures S1–S3 and Table S1 [file mmc1.pdf]

Supplementary Materials for:

Systematic Profiling of Temperature- and Retinal-Sensitive Rhodopsin Variants by Deep Mutational Scanning

Andrew G. McKee,[1†] Charles P. Kuntz,[1†] Joseph T. Ortega,[2] Hope Woods,[3,4] Victoria Most,[5] Francis J. Roushar,[1] Jens Meiler,[3,5] Beata Jastrzebska,[2] and Jonathan P. Schlebach[1*]

[1]*Department of Chemistry, Indiana University, Bloomington, Indiana, USA*
[2]*Department of Pharmacology, Case Western Reserve University, Cleveland, OH, USA*
[3]*Department of Chemistry, Vanderbilt University, Nashville, TN, USA*
[4]*Chemical and Physical Biology Program, Vanderbilt University, Nashville, TN, USA*
[5]*Institute for Drug Development, Leipzig University, Leipzig, SAC, Germany*

[†]Authors contributed equally
**Corresponding author* E-mail address: jschleba@indiana.edu

This File Includes:
Figure S1
Figure S2
Figure S3
Table S1

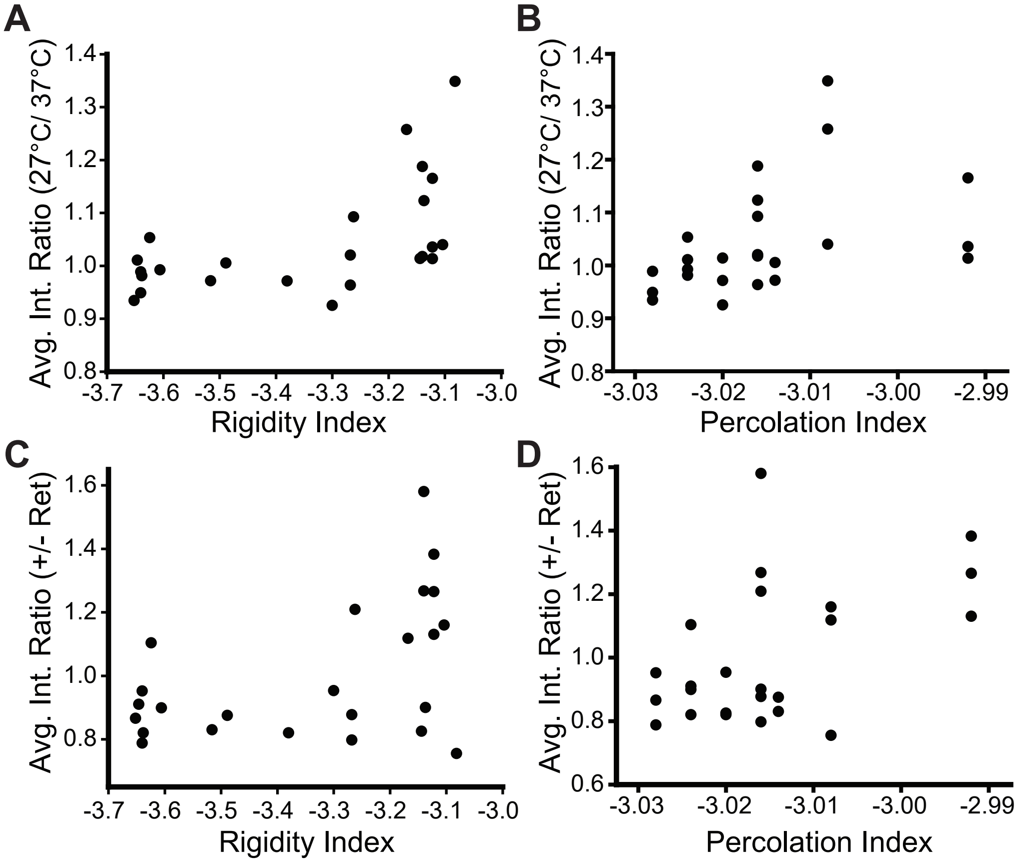

Figure S1. Structural Rigidity of TM2 Residues in Relation to the Tempertature- and Retinal- Sensitivity of TM2 Variants. The ratio of the surface immunostaining intensity at 27°C versus 37°C was averaged accross all amino acid substitutions at each TM2 residue and plotted against the corresponding rigidity index (A) or percolation index (B) values that were derived for each residue from simulations of thermal unfolding. The ratio of the surface immunostaining intensity in the presence and absence of 5 μM 9-cis-retinal was averaged accross all amino acid substitutions at each TM2 residue and plotted against the corresponding rigidity index (C) or percolation index (D) values that were derived for each residue from simulations of thermal unfolding. Lower rigidity and percolation index values, which are two measures of rigidity derived from simulations of thermal denaturation (25- 27), correspond to more structural rigidity. Percolation and rigidity index values ranged from -6 to 0, where a value of 0 corresponds to residue that completely lacks rigidity within the simulation and -6 corresponds to residues that retained rigidity the longest.

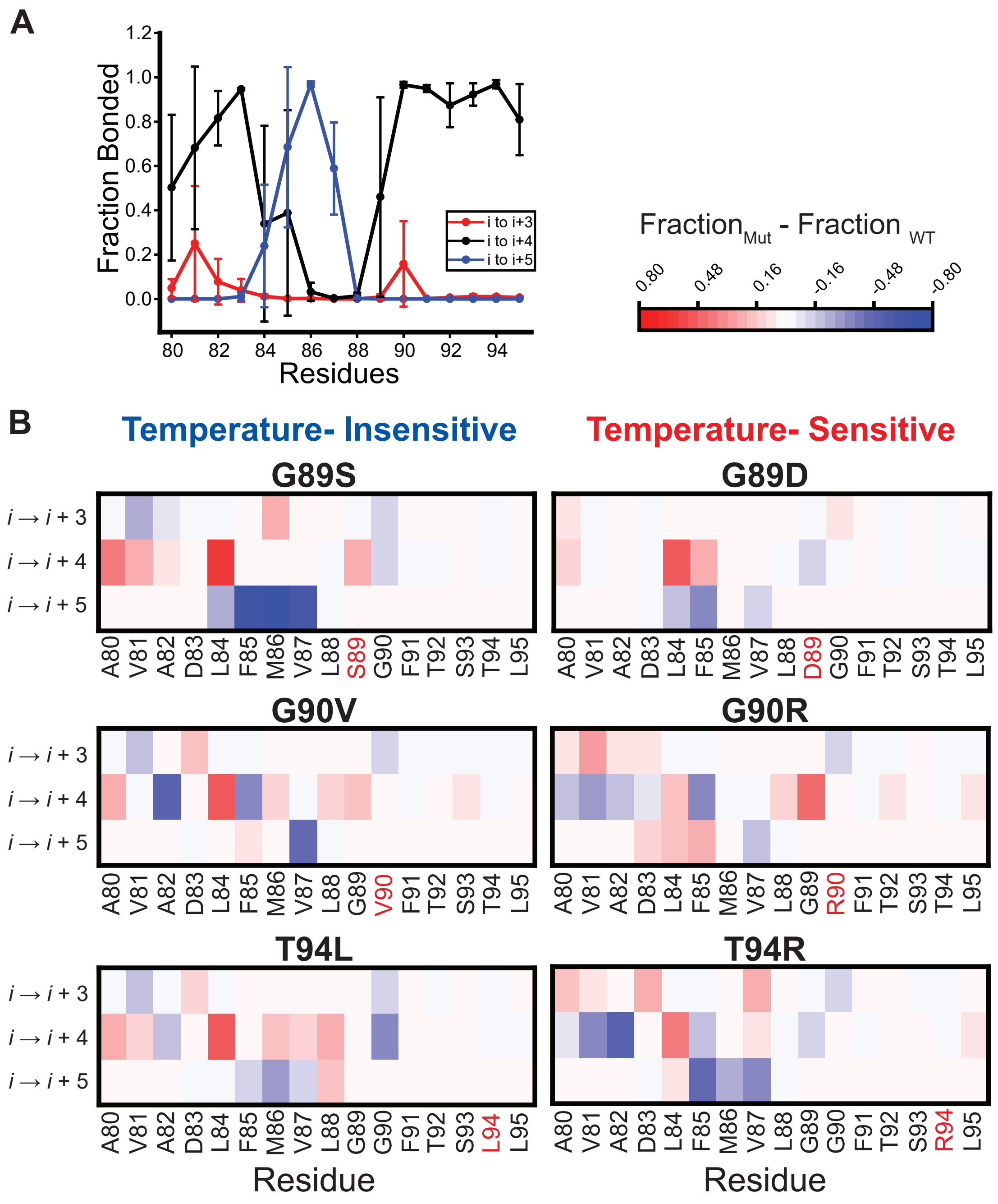

Figure S2. Backbone Hydrogen Bond Dynamics in TM2 Variants. Molecular dynamics simulations of three temperature-sensitive variants (G89D, G90R, and T94R), three temperature-insensitive variants (G89S, G90V, and T94L), and WT rhodopsin were carried out in an explicit lipid bilayer, and the fraction occupancy of backbone hydrogen bonds were calculated over the final 50 ns of each 200 ns trajectory. A) The fraction of the trajectory each residue near the kink forms 3_{10} (i to i+3), alpha (i to i+4), or pi (i to i+5) helical secondary structure is plotted for the WT protein. Values reflect the average from three replicate simulations and error bars reflect the standard deviation. B) Heat maps depict the difference between the fractional bonding between each simulated mutant and WT across three replicate simulations. The magnitude of these differences are indicated in the scale bar, for reference. These data show that most mutations in this region perturb the backbone hydrogen bonding network within the kink, but that this feature is not necessarily unique to temperature-sensitive variants.

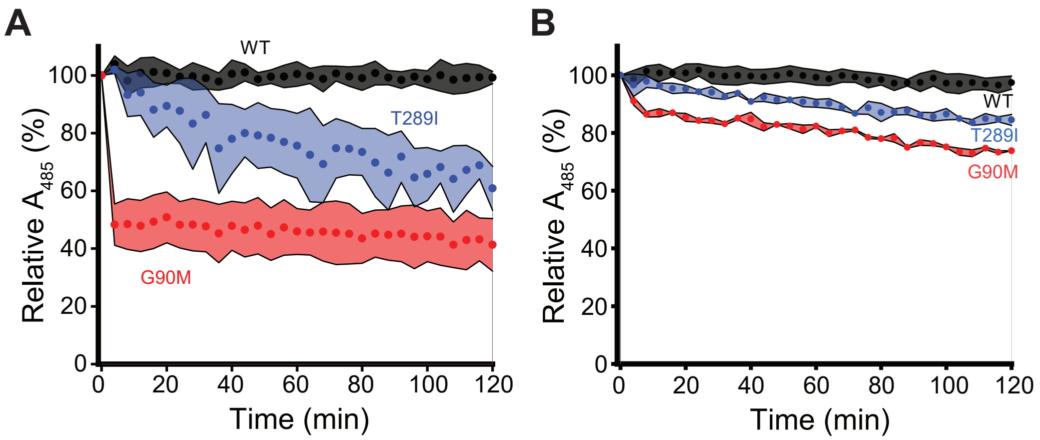

Figure S3. Kinetic Stability of Purified G90M and T289I Rhodopsins. A) WT (black), G90M (red), and T289I (blue) rhodopsins regenerated within cellular membranes prior to purification were incubated at 27°C, and the change in the absorbance at 485 nm was measured over time. Points reflect the average value and the shading reflects the bounds of the standard deviation from three experimental replicates. B) WT (black), G90M (red), and T289I (blue) rhodopsins regenerated within living cells prior to purification were incubated at 27°C, and the change in the absorbance at 485 nm was measured over time. Points reflect the average value and the shading reflects the bounds of the standard deviation from three experimental replicates.

Table S1. Temperature-Sensitivity, Retinal-Sensitivity, and Predicted Energetic Effects of Mutations in Rhodopsin

Mutation	Intensity (+Ret)/ Intensity (-Ret)	Intensity (27°C)/ Intensity (37°C)	Rosetta ΔΔG (REU)	Cα Distance to Retinal Centroid (Å)	Biological Hydrophobicity ΔΔG (kcal/ mol)	9-cis-Retinal Sensitivity *	Temperature Sensitivity *
Y 74 I	0.84	0.98	-4.880	23.41	0.004	-	-
Y 74 L	0.83	0.97	-1.960	23.41	-0.210	-	-
Y 74 F	0.80	0.98	-0.930	23.41	-0.126	-	-
Y 74 C	0.81	1.00	-1.220	23.41	0.096	-	-
Y 74 A	0.80	0.99	-1.590	23.41	0.116	-	-
Y 74 W	0.81	0.93	-2.520	23.41	-0.113	-	-
Y 74 T	0.82	0.98	-2.740	23.41	0.133	-	-
Y 74 Y	0.96	0.95	N/A	23.41	0.000	-	-
Y 74 G	0.80	0.97	0.520	23.41	0.388	-	-
Y 74 S	0.87	0.92	-1.600	23.41	0.430	-	-
Y 74 N	0.80	0.96	-2.040	23.41	0.662	-	-
Y 74 H	0.95	0.94	0.730	23.41	0.661	-	-
Y 74 P	0.89	0.98	7.230	23.41	0.527	-	-
Y 74 Q	0.82	0.94	-4.920	23.41	0.579	-	-
Y 74 R	0.79	0.99	-3.470	23.41	0.119	-	-
Y 74 E	0.78	0.99	-0.280	23.41	0.718	-	-
Y 74 K	0.80	0.95	-1.340	23.41	0.376	-	-
Y 74 D	0.83	1.07	0.840	23.41	0.492	-	-
I 75 I	0.87	0.92	N/A	19.94	0.000	-	-
I 75 L	0.85	1.02	0.170	19.94	-0.289	-	-
I 75 F	0.81	0.98	2.860	19.94	-0.155	-	-
I 75 V	0.84	0.99	1.070	19.94	0.169	-	-
I 75 M	0.85	0.96	-2.120	19.94	0.169	-	-
I 75 A	0.77	0.99	2.300	19.94	0.207	-	-
I 75 T	0.84	0.96	-1.380	19.94	0.250	-	-
I 75 Y	0.82	0.98	5.020	19.94	-0.055	-	-
I 75 G	0.81	1.01	4.140	19.94	0.610	-	-
I 75 S	0.78	0.95	-1.600	19.94	0.693	-	-
I 75 N	0.77	0.97	-0.110	19.94	1.065	-	-
I 75 H	0.80	0.95	2.840	19.94	1.049	-	-
I 75 Q	0.72	0.99	-4.470	19.94	0.958	-	-
I 75 R	1.29	1.24	3.280	19.94	0.235	+	-
I 75 E	0.77	0.96	-1.170	19.94	1.148	-	-
I 75 K	0.95	0.94	3.000	19.94	0.702	-	-
I 75 D	0.94	1.08	5.060	19.94	0.874	-	-
L 76 I	0.88	1.03	4.040	19.82	0.216	-	-
L 76 L	0.86	0.96	N/A	19.82	0.000	-	-
L 76 F	0.83	0.96	6.650	19.82	0.119	-	-
L 76 C	0.89	1.02	6.180	19.82	0.395	-	-
L 76 M	0.84	0.98	1.650	19.82	0.395	-	-
L 76 A	0.74	0.93	4.580	19.82	0.436	-	-

Mutation	Intensity (+Ret)/ Intensity (-Ret)	Intensity (27°C)/ Intensity (37°C)	Rosetta ΔΔG (REU)	C_α Distance to Retinal Centroid (Å)	Biological Hydrophobicity ΔΔG (kcal/ mol)	9-cis-Retinal Sensitivity *	Temperature Sensitivity *
L 76 W	0.83	0.97	8.030	19.82	-0.026	-	-
L 76 T	0.84	0.96	4.890	19.82	0.496	-	-
L 76 G	0.83	0.99	5.190	19.82	0.796	-	-
L 76 S	0.84	0.96	3.610	19.82	0.891	-	-
L 76 H	0.95	0.96	7.250	19.82	1.238	-	-
L 76 P	0.76	0.93	22.130	19.82	1.074	-	-
L 76 Q	0.90	0.95	3.160	19.82	1.180	-	-
L 76 R	0.90	1.23	5.800	19.82	0.499	-	-
L 76 K	0.74	0.97	5.450	19.82	0.996	-	-
L 76 D	1.19	1.04	8.720	19.82	1.149	+	-
L 77 I	0.76	1.01	-3.300	21.18	0.131	-	-
L 77 L	0.86	0.97	N/A	21.18	0.000	-	-
L 77 F	0.89	1.00	2.150	21.18	0.088	-	-
L 77 V	0.83	0.99	-0.730	21.18	0.279	-	-
L 77 A	0.85	1.00	2.430	21.18	0.317	-	-
L 77 T	0.82	0.99	1.070	21.18	0.378	-	-
L 77 H	1.32	1.08	6.360	21.18	0.895	+	-
L 77 P	0.85	1.08	16.710	21.18	0.789	-	-
L 77 Q	0.84	0.98	2.310	21.18	0.874	-	-
L 77 R	0.81	1.01	2.640	21.18	0.406	-	-
L 77 E	0.87	0.95	4.860	21.18	0.979	-	-
L 77 D	0.74	0.94	3.690	21.18	0.891	-	-
N 78 F	0.91	1.00	8.570	18.38	-1.239	-	-
N 78 C	0.82	0.97	6.020	18.38	-0.984	-	-
N 78 T	1.10	1.23	4.610	18.38	-0.804	+	-
N 78 Y	0.95	1.02	10.380	18.38	-1.058	-	-
N 78 S	0.80	0.96	3.810	18.38	-0.444	-	-
N 78 N	1.02	0.93	N/A	18.38	0.000	-	-
N 78 H	1.26	1.10	7.890	18.38	-0.058	+	-
N 78 P	1.13	1.49	20.620	18.38	-0.207	+	+
N 78 Q	1.53	1.31	5.180	18.38	-0.062	+	+
N 78 R	1.20	0.95	5.710	18.38	-0.702	+	-
N 78 E	0.99	1.06	7.040	18.38	0.083	-	-
N 78 K	1.22	0.97	6.060	18.38	-0.099	+	-
N 78 D	0.81	0.98	2.880	18.38	0.026	-	-
L 79 I	0.88	1.03	0.130	15.68	0.144	-	-
L 79 L	0.83	0.98	N/A	15.68	0.000	-	-
L 79 F	0.79	1.01	1.860	15.68	0.167	-	-
L 79 V	0.86	0.95	0.480	15.68	0.480	-	-
L 79 C	0.83	0.99	4.180	15.68	0.497	-	-
L 79 M	0.99	1.07	-1.560	15.68	0.497	-	-
L 79 A	0.79	0.98	3.260	15.68	0.596	-	-

Mutation	Intensity (+Ret)/ Intensity (-Ret)	Intensity (27°C)/ Intensity (37°C)	Rosetta ΔΔG (REU)	C_α Distance to Retinal Centroid (Å)	Biological Hydrophobicity ΔΔG (kcal/ mol)	9-cis-Retinal Sensitivity *	Temperature Sensitivity *
L 79 T	0.83	0.98	2.470	15.68	0.799	-	-
L 79 Y	0.78	0.99	3.220	15.68	0.581	-	-
L 79 S	0.77	0.99	3.820	15.68	1.227	-	-
L 79 N	0.79	1.00	-3.120	15.68	1.819	-	-
L 79 P	0.96	0.96	20.720	15.68	1.557	-	-
L 79 Q	0.97	0.97	-4.380	15.68	1.760	-	-
L 79 R	0.78	0.99	1.230	15.68	1.055	-	-
L 79 E	0.84	0.94	0.390	15.68	1.922	-	-
L 79 K	0.76	0.99	2.640	15.68	1.826	-	-
A 80 I	0.87	1.00	-1.530	17.98	-0.350	-	-
A 80 L	0.91	1.02	-1.820	17.98	-0.418	-	-
A 80 V	0.91	0.87	0.180	17.98	-0.107	-	-
A 80 C	0.82	0.98	0.540	17.98	-0.083	-	-
A 80 A	0.88	0.94	N/A	17.98	0.000	-	-
A 80 T	0.81	0.97	-2.480	17.98	0.182	-	-
A 80 Y	0.80	0.99	5.390	17.98	0.113	-	-
A 80 G	0.85	0.96	2.860	17.98	0.309	-	-
A 80 S	0.86	0.94	-3.620	17.98	0.457	-	-
A 80 N	0.83	0.93	-4.590	17.98	0.922	-	-
A 80 H	0.81	0.97	2.280	17.98	0.844	-	-
A 80 P	0.76	0.99	11.690	17.98	0.722	-	-
A 80 Q	0.69	0.90	1.470	17.98	0.890	-	-
A 80 E	0.83	0.86	2.730	17.98	1.001	-	-
A 80 K	1.30	1.02	6.590	17.98	1.030	+	-
A 80 D	0.81	0.90	0.420	17.98	1.096	-	-
V 81 I	0.83	1.02	-0.040	17.77	-0.234	-	-
V 81 L	0.85	1.07	0.470	17.77	-0.272	-	-
V 81 V	0.85	0.94	N/A	17.77	0.000	-	-
V 81 C	0.78	0.95	4.850	17.77	0.040	-	-
V 81 A	0.85	0.93	1.870	17.77	0.136	-	-
V 81 W	0.84	0.88	1.960	17.77	0.047	-	-
V 81 T	0.83	0.97	3.020	17.77	0.347	-	-
V 81 G	0.79	0.96	4.880	17.77	0.426	-	-
V 81 S	0.86	0.97	4.430	17.77	0.577	-	-
V 81 N	0.80	0.92	3.950	17.77	1.040	-	-
V 81 Q	0.94	0.91	4.990	17.77	1.020	-	-
V 81 R	1.27	0.97	5.410	17.77	0.775	+	-
V 81 E	1.00	1.19	7.120	17.77	1.116	-	-
A 82 I	0.78	0.97	6.820	13.99	-0.613	-	-
A 82 L	0.81	0.97	8.450	13.99	-0.636	-	-
A 82 F	0.85	0.95	11.880	13.99	-0.461	-	-
A 82 V	0.75	0.92	5.920	13.99	-0.272	-	-

Mutation	Intensity (+Ret)/ Intensity (-Ret)	Intensity (27°C)/ Intensity (37°C)	Rosetta ΔΔG (REU)	C_α Distance to Retinal Centroid (Å)	Biological Hydrophobicity ΔΔG (kcal/ mol)	9-cis-Retinal Sensitivity *	Temperature Sensitivity *
A 82 C	0.80	0.95	5.430	13.99	-0.175	-	-
A 82 M	0.80	0.96	1.580	13.99	-0.176	-	-
A 82 A	0.85	0.96	N/A	13.99	0.000	-	-
A 82 W	0.86	0.98	12.510	13.99	-0.007	-	-
A 82 T	0.83	0.96	1.790	13.99	0.376	-	-
A 82 Y	0.83	0.94	13.690	13.99	0.441	-	-
A 82 G	0.81	1.00	0.750	13.99	0.434	-	-
A 82 S	0.82	0.95	0.810	13.99	0.677	-	-
A 82 H	0.85	0.94	9.780	13.99	1.252	-	-
A 82 P	0.77	0.99	16.900	13.99	1.100	-	-
A 82 Q	0.79	0.90	9.450	13.99	1.371	-	-
A 82 R	0.70	0.98	9.160	13.99	1.243	-	-
A 82 E	0.77	0.95	9.720	13.99	1.496	-	-
A 82 K	0.80	0.96	7.720	13.99	1.796	-	-
A 82 D	0.74	0.95	9.680	13.99	1.804	-	-
D 83 I	0.74	0.97	2.070	13.95	-2.205	-	-
D 83 L	0.77	1.00	1.240	13.95	-2.201	-	-
D 83 F	0.78	0.97	3.220	13.95	-2.058	-	-
D 83 V	0.78	0.96	4.520	13.95	-1.953	-	-
D 83 C	0.77	1.01	1.870	13.95	-1.839	-	-
D 83 A	0.82	0.94	0.930	13.95	-1.671	-	-
D 83 W	0.76	1.00	-1.250	13.95	-1.600	-	-
D 83 T	0.74	0.94	1.800	13.95	-1.314	-	-
D 83 Y	0.86	0.98	4.080	13.95	-1.231	-	-
D 83 G	0.77	0.99	0.350	13.95	-1.309	-	-
D 83 S	0.84	0.97	-1.560	13.95	-1.090	-	-
D 83 N	0.87	0.97	-5.260	13.95	-0.445	-	-
D 83 H	0.82	0.96	-0.260	13.95	-0.573	-	-
D 83 R	0.82	0.98	4.640	13.95	-0.362	-	-
D 83 E	0.79	0.93	4.030	13.95	-0.344	-	-
D 83 K	0.74	0.99	12.340	13.95	0.023	-	-
D 83 D	0.96	1.02	N/A	13.95	0.000	-	-
L 84 I	0.94	0.95	0.700	16.76	-0.016	-	-
L 84 L	0.88	0.99	N/A	16.76	0.000	-	-
L 84 F	0.85	0.99	6.250	16.76	0.112	-	-
L 84 V	0.95	0.97	1.950	16.76	0.158	-	-
L 84 C	0.87	0.99	5.520	16.76	0.268	-	-
L 84 M	1.14	1.24	2.230	16.76	0.267	+	-
L 84 A	0.87	0.94	4.630	16.76	0.410	-	-
L 84 T	0.89	0.95	1.290	16.76	0.697	-	-
L 84 Y	1.01	1.29	6.510	16.76	0.763	-	+
L 84 G	0.82	0.93	6.710	16.76	0.677	-	-

Mutation	Intensity (+Ret)/ Intensity (-Ret)	Intensity (27°C)/ Intensity (37°C)	Rosetta ΔΔG (REU)	C_α Distance to Retinal Centroid (Å)	Biological Hydrophobicity ΔΔG (kcal/ mol)	9-cis-Retinal Sensitivity *	Temperature Sensitivity *
L 84 S	0.88	0.95	2.410	16.76	0.841	-	-
L 84 N	0.85	0.94	5.660	16.76	1.313	-	-
L 84 H	0.84	0.97	8.980	16.76	1.217	-	-
L 84 P	1.05	1.27	15.790	16.76	1.128	-	-
L 84 Q	0.86	0.93	3.580	16.76	1.316	-	-
L 84 R	1.24	0.98	5.000	16.76	1.492	+	-
L 84 E	0.91	0.90	4.170	16.76	1.385	-	-
L 84 D	1.16	0.78	8.940	16.76	1.665	+	-
F 85 I	0.78	1.00	1.250	14.87	-0.174	-	-
F 85 L	0.79	0.97	0.150	14.87	-0.145	-	-
F 85 F	0.83	0.95	N/A	14.87	0.000	-	-
F 85 V	0.80	0.97	1.680	14.87	0.034	-	-
F 85 C	1.03	1.06	2.330	14.87	0.195	-	-
F 85 M	0.71	1.06	-1.310	14.87	0.194	-	-
F 85 A	0.76	0.95	1.320	14.87	0.393	-	-
F 85 W	0.89	0.96	1.350	14.87	0.533	-	-
F 85 T	0.81	0.95	1.160	14.87	0.795	-	-
F 85 Y	0.96	1.05	0.630	14.87	0.886	-	-
F 85 G	0.82	0.94	3.570	14.87	0.751	-	-
F 85 S	0.78	0.98	2.580	14.87	0.978	-	-
F 85 N	0.86	0.99	1.620	14.87	1.641	-	-
F 85 H	0.88	0.98	2.880	14.87	1.504	-	-
F 85 P	0.78	0.98	13.200	14.87	1.382	-	-
F 85 R	1.20	1.27	3.340	14.87	1.983	+	-
F 85 K	0.85	0.99	3.570	14.87	2.212	-	-
F 85 D	0.76	0.98	4.800	14.87	2.153	-	-
M 86 I	0.82	0.99	2.800	12.09	-0.486	-	-
M 86 L	0.82	0.99	0.010	12.09	-0.447	-	-
M 86 F	0.97	0.94	5.320	12.09	-0.255	-	-
M 86 V	0.78	0.94	4.180	12.09	-0.210	-	-
M 86 C	0.86	0.97	3.010	12.09	0.001	-	-
M 86 A	0.79	0.92	2.960	12.09	0.257	-	-
M 86 T	0.82	0.94	3.510	12.09	0.763	-	-
M 86 Y	0.80	1.00	8.220	12.09	0.875	-	-
M 86 G	0.85	0.97	4.320	12.09	0.708	-	-
M 86 S	0.83	0.99	3.770	12.09	0.988	-	-
M 86 N	0.86	1.01	3.550	12.09	1.780	-	-
M 86 H	0.86	0.94	5.990	12.09	1.619	-	-
M 86 P	0.70	0.99	15.760	12.09	1.474	-	-
M 86 Q	0.69	0.99	4.400	12.09	1.788	-	-
M 86 R	0.69	0.98	1.040	12.09	2.175	-	-
M 86 E	0.80	0.91	6.020	12.09	1.897	-	-

Mutation	Intensity (+Ret)/ Intensity (-Ret)	Intensity (27°C)/ Intensity (37°C)	Rosetta ΔΔG (REU)	C_α Distance to Retinal Centroid (Å)	Biological Hydrophobicity ΔΔG (kcal/ mol)	9-cis-Retinal Sensitivity *	Temperature Sensitivity *
M 86 K	0.69	0.94	1.260	12.09	2.437	-	-
M 86 D	0.94	0.97	6.080	12.09	2.370	-	-
V 87 I	0.84	0.97	-2.300	15.04	-0.220	-	-
V 87 L	1.00	1.07	-1.030	15.04	-0.200	-	-
V 87 V	0.90	0.94	N/A	15.04	0.000	-	-
V 87 C	0.91	0.97	2.120	15.04	0.140	-	-
V 87 M	0.96	1.03	-0.450	15.04	0.139	-	-
V 87 A	0.97	0.98	2.530	15.04	0.322	-	-
V 87 T	0.80	0.94	1.580	15.04	0.699	-	-
V 87 S	1.00	0.97	3.820	15.04	0.891	-	-
V 87 N	1.88	1.29	0.880	15.04	1.533	+	+
V 87 H	1.17	1.07	2.970	15.04	1.402	+	-
V 87 P	1.69	1.72	18.290	15.04	1.280	+	+
V 87 Q	0.99	1.03	2.550	15.04	1.536	-	-
V 87 E	1.06	0.98	3.370	15.04	1.631	-	-
V 87 D	1.57	1.38	4.600	15.04	2.011	+	+
L 88 I	0.86	0.96	-0.560	17.32	-0.004	-	-
L 88 L	0.84	0.98	N/A	17.32	0.000	-	-
L 88 F	0.95	1.04	1.270	17.32	0.111	-	-
L 88 V	0.88	1.00	-0.370	17.32	0.192	-	-
L 88 M	0.85	0.98	0.040	17.32	0.279	-	-
L 88 A	0.93	1.08	1.770	17.32	0.407	-	-
L 88 W	1.14	0.89	2.090	17.32	0.461	+	-
L 88 T	0.91	1.01	1.730	17.32	0.673	-	-
L 88 G	0.93	0.98	3.860	17.32	0.677	-	-
L 88 S	0.91	0.99	3.300	17.32	0.838	-	-
L 88 H	1.26	0.99	6.220	17.32	1.213	+	-
L 88 P	1.85	1.25	21.750	17.32	1.120	+	-
L 88 Q	1.17	1.10	4.440	17.32	1.303	+	-
L 88 R	1.19	0.98	3.940	17.32	1.366	+	-
L 88 E	1.36	1.15	7.320	17.32	1.379	+	-
L 88 K	1.47	1.07	4.820	17.32	1.647	+	-
L 88 D	1.00	1.03	8.480	17.32	1.629	-	-
G 89 I	0.70	0.99	-1.430	15.32	-0.964	-	-
G 89 L	0.74	0.97	0.990	15.32	-0.986	-	-
G 89 F	0.75	0.98	-0.660	15.32	-0.827	-	-
G 89 V	0.95	0.96	-0.030	15.32	-0.654	-	-
G 89 A	0.87	1.01	2.660	15.32	-0.404	-	-
G 89 W	0.82	1.18	0.930	15.32	-0.410	-	-
G 89 T	0.92	1.06	0.620	15.32	-0.055	-	-
G 89 G	0.92	0.87	N/A	15.32	0.000	-	-
G 89 S	1.03	1.07	2.730	15.32	0.227	-	-

Mutation	Intensity (+Ret)/ Intensity (-Ret)	Intensity (27°C)/ Intensity (37°C)	Rosetta ΔΔG (REU)	C_α Distance to Retinal Centroid (Å)	Biological Hydrophobicity ΔΔG (kcal/ mol)	9-cis-Retinal Sensitivity *	Temperature Sensitivity *
G 89 N	1.16	1.03	4.330	15.32	0.898	+	-
G 89 H	1.58	1.48	6.370	15.32	0.771	+	+
G 89 P	0.85	1.02	12.900	15.32	0.627	-	-
G 89 Q	1.21	1.24	4.680	15.32	0.885	+	-
G 89 E	0.93	1.35	7.890	15.32	1.004	-	+
G 89 D	1.01	1.57	8.400	15.32	1.299	-	+
G 90 I	0.99	1.39	2.860	12.89	-0.972	-	+
G 90 L	1.21	1.49	10.200	12.89	-1.029	+	+
G 90 V	0.72	1.04	3.600	12.89	-0.628	-	-
G 90 M	2.03	1.46	-2.170	12.89	-0.299	+	+
G 90 A	0.84	1.02	-3.150	12.89	-0.427	-	-
G 90 T	0.80	1.06	-4.240	12.89	-0.116	-	-
G 90 Y	1.85	1.59	0.180	12.89	-0.114	+	+
G 90 G	0.83	0.99	N/A	12.89	0.000	-	-
G 90 S	0.76	1.01	-3.030	12.89	0.221	-	-
G 90 R	0.77	2.38	3.950	12.89	0.507	-	+
G 90 D	0.68	0.95	5.240	12.89	1.209	-	-
F 91 I	1.11	1.11	2.040	14.09	-0.045	+	-
F 91 L	1.00	1.08	0.430	14.09	-0.109	-	-
F 91 F	0.82	0.96	N/A	14.09	0.000	-	-
F 91 V	1.08	1.09	1.590	14.09	0.183	+	-
F 91 C	1.13	0.99	3.020	14.09	0.205	+	-
F 91 W	0.81	0.97	5.310	14.09	0.097	-	-
F 91 T	0.98	1.04	-0.050	14.09	0.452	-	-
F 91 Y	0.75	1.00	1.080	14.09	0.388	-	-
F 91 G	0.79	0.99	4.430	14.09	0.570	-	-
F 91 S	0.90	1.01	3.620	14.09	0.707	-	-
F 91 N	0.80	1.13	2.370	14.09	1.137	-	-
F 91 H	0.75	0.96	2.250	14.09	1.065	-	-
F 91 P	0.68	1.10	4.770	14.09	0.952	-	-
F 91 E	0.76	0.96	4.550	14.09	1.210	-	-
F 91 D	0.77	1.00	5.070	14.09	1.297	-	-
T 92 I	1.28	1.32	-2.940	17.64	-0.448	+	+
T 92 L	0.92	1.02	-2.640	17.64	-0.545	-	-
T 92 F	1.08	1.00	-1.600	17.64	-0.433	+	-
T 92 V	0.99	1.05	-2.540	17.64	-0.220	-	-
T 92 A	1.05	1.13	-0.640	17.64	-0.141	-	-
T 92 W	1.12	1.05	-1.530	17.64	-0.441	+	-
T 92 T	0.88	0.98	N/A	17.64	0.000	-	-
T 92 G	0.93	1.00	1.280	17.64	0.167	-	-
T 92 S	0.92	0.99	1.930	17.64	0.300	-	-
T 92 N	1.32	1.08	0.850	17.64	0.728	+	-

Mutation	Intensity (+Ret)/ Intensity (-Ret)	Intensity (27°C)/ Intensity (37°C)	Rosetta ΔΔG (REU)	C_α Distance to Retinal Centroid (Å)	Biological Hydrophobicity ΔΔG (kcal/ mol)	9-cis-Retinal Sensitivity *	Temperature Sensitivity *
T 92 P	1.34	1.18	5.460	17.64	0.537	+	-
T 92 Q	1.58	1.12	-0.180	17.64	0.685	+	-
T 92 R	1.18	1.41	-4.020	17.64	0.178	+	+
T 92 E	3.58	1.55	2.700	17.64	0.805	+	+
T 92 K	1.60	1.19	1.010	17.64	0.734	+	-
S 93 I	0.92	0.92	1.970	16.65	-0.982	-	-
S 93 L	0.80	0.98	-0.270	16.65	-1.167	-	-
S 93 F	0.88	0.95	2.010	16.65	-1.008	-	-
S 93 V	0.81	0.96	1.880	16.65	-0.685	-	-
S 93 C	0.85	0.95	3.710	16.65	-0.678	-	-
S 93 A	1.51	0.86	3.310	16.65	-0.599	+	-
S 93 T	0.86	0.96	3.300	16.65	-0.449	-	-
S 93 G	0.92	0.97	3.790	16.65	-0.162	-	-
S 93 S	0.85	0.92	N/A	16.65	0.000	-	-
S 93 P	0.77	0.96	9.830	16.65	0.290	-	-
S 93 Q	0.85	0.92	1.500	16.65	0.464	-	-
S 93 R	2.24	1.43	0.410	16.65	-0.320	+	+
S 93 D	1.54	0.86	4.220	16.65	0.569	+	-
T 94 L	0.82	1.04	9.100	14.70	-0.528	-	-
T 94 F	1.05	1.06	9.620	14.70	-0.406	-	-
T 94 C	0.93	1.08	4.860	14.70	-0.138	-	-
T 94 A	0.98	0.99	8.640	14.70	-0.087	-	-
T 94 W	1.26	0.90	14.790	14.70	-0.562	+	-
T 94 T	0.94	0.97	N/A	14.70	0.000	-	-
T 94 Y	1.26	1.23	12.090	14.70	-0.315	+	-
T 94 S	0.99	0.99	2.150	14.70	0.385	-	-
T 94 N	0.71	1.01	2.950	14.70	0.799	-	-
T 94 P	1.16	1.15	14.370	14.70	0.597	+	-
T 94 R	1.18	1.41	3.410	14.70	0.040	+	+
T 94 E	0.71	0.94	8.280	14.70	0.881	-	-
T 94 K	0.87	1.43	6.110	14.70	0.605	-	+
L 95 I	0.90	1.01	6.990	17.76	0.151	-	-
L 95 L	0.95	1.00	N/A	17.76	0.000	-	-
L 95 F	0.77	0.94	0.050	17.76	0.083	-	-
L 95 C	0.82	0.91	4.160	17.76	0.273	-	-
L 95 M	0.87	0.96	-2.360	17.76	0.273	-	-
L 95 A	0.76	0.91	2.990	17.76	0.302	-	-
L 95 W	0.91	1.09	0.670	17.76	-0.019	-	-
L 95 G	0.81	0.93	5.010	17.76	0.544	-	-
L 95 S	0.81	0.90	3.820	17.76	0.607	-	-
L 95 H	1.69	1.12	3.330	17.76	0.836	+	-
L 95 P	1.33	1.69	10.090	17.76	0.728	+	+

Mutation	Intensity (+Ret)/ Intensity (-Ret)	Intensity (27°C)/ Intensity (37°C)	Rosetta ΔΔG (REU)	C_α Distance to Retinal Centroid (Å)	Biological Hydrophobicity ΔΔG (kcal/ mol)	9-cis-Retinal Sensitivity *	Temperature Sensitivity *
L 95 Q	1.73	1.13	2.150	17.76	0.798	+	-
L 95 R	0.85	1.01	0.780	17.76	0.345	-	-
L 95 K	1.28	0.96	2.430	17.76	0.677	+	-
L 95 D	1.23	0.97	5.980	17.76	0.778	+	-
Y 96 I	0.76	0.94	-2.020	20.20	0.045	-	-
Y 96 L	0.85	0.96	-1.650	20.20	-0.196	-	-
Y 96 F	0.88	0.95	-0.210	20.20	-0.084	-	-
Y 96 V	0.77	0.94	-1.240	20.20	0.189	-	-
Y 96 C	0.93	0.92	1.650	20.20	0.189	-	-
Y 96 A	0.90	0.95	-0.350	20.20	0.221	-	-
Y 96 T	0.85	0.91	-0.750	20.20	0.258	-	-
Y 96 Y	1.02	0.95	N/A	20.20	0.000	-	-
Y 96 G	0.90	1.04	0.920	20.20	0.567	-	-
Y 96 S	0.95	0.97	-0.020	20.20	0.639	-	-
Y 96 N	1.28	0.99	-2.340	20.20	0.966	+	-
Y 96 H	0.97	0.92	1.570	20.20	0.952	-	-
Y 96 P	1.00	0.98	5.220	20.20	0.788	-	-
Y 96 R	2.81	1.72	-7.670	20.20	0.245	+	+
Y 96 E	1.59	0.94	1.190	20.20	1.039	+	-
Y 96 K	1.37	0.86	-0.500	20.20	0.647	+	-
Y 96 D	1.25	0.98	0.810	20.20	0.797	+	-
T 97 I	1.48	1.31	4.100	18.55	-0.185	+	+
T 97 C	0.78	0.97	0.790	18.55	-0.053	-	-
T 97 A	0.83	0.92	2.550	18.55	-0.025	-	-
T 97 T	1.11	1.02	N/A	18.55	0.000	+	-
T 97 G	0.74	0.98	6.680	18.55	0.358	-	-
T 97 S	1.00	0.97	1.280	18.55	0.416	-	-
T 97 N	1.19	1.03	-0.940	18.55	0.734	+	-
T 97 P	1.04	1.02	14.120	18.55	0.550	-	-
T 97 R	1.15	1.68	4.770	18.55	-0.020	+	+
T 97 K	1.20	1.12	10.010	18.55	0.342	+	-
F 287 I	0.69	0.87	-0.940	15.07	0.106	-	-
F 287 L	0.67	0.91	-1.140	15.07	-0.091	-	-
F 287 F	0.76	0.91	nan	15.07	0.000	-	-
F 287 V	0.70	0.88	-1.200	15.07	0.224	-	-
F 287 C	0.86	0.85	1.680	15.07	0.224	-	-
F 287 A	0.66	0.93	-0.350	15.07	0.250	-	-
F 287 T	0.69	0.90	-0.780	15.07	0.280	-	-
F 287 Y	0.78	0.78	-1.690	15.07	0.069	-	-
F 287 G	0.74	0.75	-0.070	15.07	0.538	-	-
F 287 S	0.66	0.90	-0.540	15.07	0.598	-	-
F 287 N	0.50	1.18	-0.630	15.07	0.877	-	+

Mutation	Intensity (+Ret)/ Intensity (-Ret)	Intensity (27°C)/ Intensity (37°C)	Rosetta ΔΔG (REU)	C_α Distance to Retinal Centroid (Å)	Biological Hydrophobicity ΔΔG (kcal/ mol)	9-cis-Retinal Sensitivity *	Temperature Sensitivity *
F 287 H	0.78	0.88	0.020	15.07	0.865	-	-
F 287 P	0.66	0.86	12.120	15.07	0.724	-	-
F 287 Q	0.67	0.94	0.060	15.07	0.796	-	-
F 287 R	0.67	0.86	-2.820	15.07	0.270	-	-
F 287 E	0.77	0.99	1.740	15.07	0.940	-	-
F 287 K	0.71	0.91	-0.930	15.07	0.605	-	-
M 288 I	0.68	0.77	-1.000	11.54	-0.210	-	-
M 288 L	0.80	0.74	-2.920	11.54	-0.468	-	-
M 288 F	0.73	0.81	2.260	11.54	-0.326	-	-
M 288 V	0.91	0.74	1.910	11.54	-0.001	-	-
M 288 C	0.81	0.92	0.920	11.54	0.000	-	-
M 288 A	0.75	0.81	-0.260	11.54	0.049	-	-
M 288 T	0.69	0.84	-4.290	11.54	0.118	-	-
M 288 G	0.88	0.96	0.740	11.54	0.465	-	-
M 288 S	0.79	0.91	-1.890	11.54	0.574	-	-
M 288 N	0.81	1.05	-4.350	11.54	1.000	-	+
M 288 H	0.83	0.87	-2.450	11.54	0.968	-	-
M 288 P	0.77	0.87	2.060	11.54	0.782	-	-
M 288 Q	0.80	0.74	-0.920	11.54	0.902	-	-
M 288 R	0.88	0.86	-0.640	11.54	0.122	-	-
M 288 E	0.90	0.75	1.890	11.54	1.088	-	-
T 289 I	0.91	1.18	7.000	12.58	-0.351	-	+
T 289 L	0.75	0.97	3.470	12.58	-0.532	-	-
T 289 F	0.69	0.97	5.390	12.58	-0.411	-	-
T 289 A	0.66	0.97	4.740	12.58	-0.088	-	-
T 289 Y	0.61	0.92	5.460	12.58	-0.319	-	-
T 289 G	0.68	0.92	4.300	12.58	0.277	-	-
T 289 S	0.71	0.94	3.600	12.58	0.394	-	-
T 289 N	0.70	0.99	5.000	12.58	0.820	-	-
T 289 H	0.62	0.94	4.850	12.58	0.775	-	-
T 289 Q	0.70	0.88	4.570	12.58	0.743	-	-
T 289 R	1.14	1.67	3.840	12.58	0.041	+	+
T 289 E	0.64	1.12	8.870	12.58	0.905	-	+
T 289 K	0.96	1.80	5.720	12.58	0.620	-	+
T 289 D	0.98	1.03	7.280	12.58	0.769	+	-
I 290 I	0.73	0.83	nan	13.10	0.000	-	-
I 290 L	0.76	0.86	-0.770	13.10	-0.108	-	-
I 290 F	0.84	0.93	-0.350	13.10	-0.015	-	-
I 290 V	0.77	0.97	0.450	13.10	0.173	-	-
I 290 A	0.75	0.93	1.020	13.10	0.224	-	-
I 290 W	0.69	0.90	-0.220	13.10	-0.096	-	-
I 290 T	0.74	0.86	1.550	13.10	0.311	-	-

Mutation	Intensity (+Ret)/ Intensity (-Ret)	Intensity (27°C)/ Intensity (37°C)	Rosetta ΔΔG (REU)	C_α Distance to Retinal Centroid (Å)	Biological Hydrophobicity ΔΔG (kcal/ mol)	9-cis-Retinal Sensitivity *	Temperature Sensitivity *
I 290 G	0.83	0.84	-4.300	13.10	0.478	-	-
I 290 N	1.21	1.54	0.060	13.10	0.884	+	+
I 290 P	0.86	0.96	9.510	13.10	0.740	-	-
I 290 R	1.08	1.25	-0.750	13.10	0.386	+	+
I 290 D	0.93	0.94	1.630	13.10	0.902	-	-
P 291 I	0.71	0.83	-2.960	9.98	-1.094	-	-
P 291 L	0.75	0.87	-2.570	9.98	-1.198	-	-
P 291 F	0.86	0.78	-3.970	9.98	-1.077	-	-
P 291 V	0.81	0.84	-4.020	9.98	-0.846	-	-
P 291 C	0.80	0.83	-1.890	9.98	-0.833	-	-
P 291 M	0.90	0.85	-4.380	9.98	-0.833	-	-
P 291 A	0.83	0.84	-2.480	9.98	-0.759	-	-
P 291 W	0.85	0.66	-3.810	9.98	-1.087	-	-
P 291 T	0.81	0.83	-4.110	9.98	-0.603	-	-
P 291 P	0.73	0.89	nan	9.98	0.000	-	-
P 291 Q	0.89	1.00	-2.410	9.98	0.168	-	-
P 291 R	0.79	0.91	-1.960	9.98	-0.404	-	-
A 292 L	0.71	1.07	-2.410	7.84	-0.621	-	+
A 292 F	0.98	1.31	-0.510	7.84	-0.448	+	+
A 292 V	0.74	0.96	1.010	7.84	-0.157	-	-
A 292 A	0.70	0.88	nan	7.84	0.000	-	-
A 292 W	0.72	1.15	-0.870	7.84	-0.294	-	+
A 292 T	0.72	0.94	0.410	7.84	0.265	-	-
A 292 G	0.68	0.95	2.210	7.84	0.448	-	-
A 292 S	0.79	0.71	1.720	7.84	0.659	-	-
A 292 N	0.78	0.82	0.310	7.84	1.303	-	-
A 292 Q	0.75	0.87	-2.010	7.84	1.260	-	-
A 292 R	0.69	0.84	-6.340	7.84	0.698	-	-
A 292 E	0.81	1.01	2.700	7.84	1.410	-	-
F 293 I	0.64	0.94	-2.500	10.55	-0.082	-	-
F 293 L	0.69	0.97	-4.220	10.55	-0.124	-	-
F 293 F	0.88	0.91	nan	10.55	0.000	-	-
F 293 V	0.70	0.95	-0.180	10.55	0.177	-	-
F 293 C	0.63	0.94	-0.300	10.55	0.222	-	-
F 293 M	0.73	0.97	-2.860	10.55	0.222	-	-
F 293 A	0.68	1.01	-0.750	10.55	0.330	-	-
F 293 W	0.80	1.08	1.480	10.55	0.230	-	+
F 293 T	0.69	0.91	2.160	10.55	0.572	-	-
F 293 G	0.65	0.91	1.230	10.55	0.663	-	-
F 293 S	0.64	0.92	2.970	10.55	0.840	-	-
F 293 N	0.69	0.92	-0.750	10.55	1.392	-	-
F 293 H	0.67	0.89	3.580	10.55	1.291	-	-

Mutation	Intensity (+Ret)/ Intensity (-Ret)	Intensity (27°C)/ Intensity (37°C)	Rosetta ΔΔG (REU)	C_α Distance to Retinal Centroid (Å)	Biological Hydrophobicity ΔΔG (kcal/ mol)	9-cis-Retinal Sensitivity *	Temperature Sensitivity *
F 293 P	0.75	1.06	16.520	10.55	1.160	-	+
F 293 Q	0.71	0.96	0.730	10.55	1.369	-	-
F 293 R	1.05	1.43	1.090	10.55	1.074	+	+
F 293 E	0.67	0.91	3.580	10.55	1.484	-	-
F 293 K	0.79	1.04	0.540	10.55	1.633	-	-
F 293 D	0.74	0.94	2.330	10.55	1.674	-	-
F 294 I	0.70	0.87	-2.320	10.98	-0.099	-	-
F 294 L	0.73	0.92	-3.220	10.98	-0.114	-	-
F 294 F	0.89	0.70	nan	10.98	0.000	-	-
F 294 V	0.72	0.85	-1.180	10.98	0.124	-	-
F 294 C	0.74	0.89	1.650	10.98	0.189	-	-
F 294 M	0.73	0.85	-0.590	10.98	0.188	-	-
F 294 A	0.68	0.78	1.520	10.98	0.304	-	-
F 294 W	0.77	0.85	-3.070	10.98	0.300	-	-
F 294 T	0.73	0.85	0.820	10.98	0.557	-	-
F 294 Y	0.81	0.83	1.940	10.98	0.602	-	-
F 294 G	0.77	0.92	3.250	10.98	0.597	-	-
F 294 S	0.77	0.84	2.800	10.98	0.764	-	-
F 294 N	0.76	0.79	2.820	10.98	1.278	-	-
F 294 H	0.80	0.71	3.940	10.98	1.178	-	-
F 294 P	0.67	0.92	5.760	10.98	1.066	-	-
F 294 Q	0.79	0.81	-1.220	10.98	1.267	-	-
F 294 R	0.97	0.92	-0.170	10.98	1.171	+	-
F 294 E	1.00	0.86	4.670	10.98	1.363	+	-
F 294 K	0.95	0.86	2.510	10.98	1.599	-	-
A 295 I	0.63	0.99	2.460	7.71	-0.646	-	-
A 295 L	0.66	0.88	-2.950	7.71	-0.640	-	-
A 295 V	0.67	0.91	1.140	7.71	-0.340	-	-
A 295 T	0.73	0.84	-3.520	7.71	0.424	-	-
A 295 G	0.71	0.94	0.810	7.71	0.430	-	-
A 295 S	0.73	0.89	-1.600	7.71	0.685	-	-
A 295 R	0.66	0.93	2.260	7.71	1.515	-	-
A 295 D	0.89	0.93	6.620	7.71	1.917	-	-
K 296 F	0.57	0.88	2.220	9.12	-2.123	-	-
K 296 V	1.05	2.57	1.260	9.12	-2.067	+	+
K 296 C	0.77	1.45	2.500	9.12	-1.933	-	+
K 296 M	0.65	0.98	-1.790	9.12	-1.934	-	-
K 296 A	0.68	1.50	2.910	9.12	-1.756	-	+
K 296 W	0.59	0.95	-0.200	9.12	-1.644	-	-
K 296 T	0.89	2.44	1.430	9.12	-1.387	-	+
K 296 Y	0.64	0.94	-2.160	9.12	-1.299	-	-
K 296 G	0.61	1.23	4.100	9.12	-1.413	-	+

Mutation	Intensity (+Ret)/ Intensity (-Ret)	Intensity (27°C)/ Intensity (37°C)	Rosetta ΔΔG (REU)	C_α Distance to Retinal Centroid (Å)	Biological Hydrophobicity ΔΔG (kcal/ mol)	9-cis-Retinal Sensitivity *	Temperature Sensitivity *
K 296 S	0.63	1.19	1.360	9.12	-1.195	-	+
K 296 N	0.54	1.03	2.370	9.12	-0.543	-	-
K 296 H	0.62	0.94	4.290	9.12	-0.677	-	-
K 296 Q	0.59	0.98	2.400	9.12	-0.539	-	-
K 296 R	0.64	0.87	2.470	9.12	-0.291	-	-
K 296 E	0.71	1.34	4.500	9.12	-0.441	-	+
K 296 K	0.77	0.84	nan	9.12	0.000	-	-
K 296 D	0.54	1.06	4.890	9.12	-0.048	-	+
S 297 L	0.69	0.88	-6.690	11.91	-0.840	-	-
S 297 F	0.72	0.88	0.330	11.91	-0.728	-	-
S 297 V	0.69	0.93	-2.950	11.91	-0.701	-	-
S 297 A	0.68	0.89	-2.380	11.91	-0.429	-	-
S 297 T	0.75	0.85	-3.290	11.91	-0.133	-	-
S 297 G	0.67	0.84	1.400	11.91	-0.165	-	-
S 297 S	0.74	0.89	nan	11.91	0.000	-	-
S 297 P	0.66	0.87	4.820	11.91	0.288	-	-
S 297 R	0.96	1.11	-9.570	11.91	0.712	+	+
S 297 E	0.86	0.91	-2.900	11.91	0.543	-	-
A 298 I	0.66	0.80	-3.430	10.16	-0.534	-	-
A 298 L	0.72	0.85	-1.660	10.16	-0.507	-	-
A 298 F	0.84	0.87	-1.400	10.16	-0.371	-	-
A 298 C	0.72	0.88	1.560	10.16	-0.188	-	-
A 298 A	0.75	0.92	nan	10.16	0.000	-	-
A 298 W	0.73	0.83	-4.090	10.16	0.133	-	-
A 298 T	0.77	0.88	-4.930	10.16	0.384	-	-
A 298 Y	0.77	0.82	-1.000	10.16	0.472	-	-
A 298 G	0.72	0.86	2.000	10.16	0.342	-	-
A 298 N	0.80	0.85	-0.800	10.16	1.213	-	-
A 298 H	0.78	0.84	1.740	10.16	1.077	-	-
A 298 P	0.73	0.88	5.150	10.16	0.956	-	-
A 298 R	0.67	0.89	-2.600	10.16	1.554	-	-
A 298 K	0.71	0.94	1.060	10.16	1.784	-	-
A 299 I	0.70	0.86	2.240	12.22	-0.726	-	-
A 299 L	0.72	0.82	5.620	12.22	-0.698	-	-
A 299 F	0.71	0.79	6.090	12.22	-0.507	-	-
A 299 V	0.72	0.87	-1.220	12.22	-0.428	-	-
A 299 C	0.78	0.83	0.470	12.22	-0.241	-	-
A 299 M	0.75	0.88	2.390	12.22	-0.242	-	-
A 299 A	0.71	0.82	nan	12.22	0.000	-	-
A 299 T	0.73	0.90	-0.410	12.22	0.485	-	-
A 299 Y	0.69	0.96	9.250	12.22	0.596	-	-
A 299 G	0.77	0.88	1.740	12.22	0.451	-	-

Mutation	Intensity (+Ret)/ Intensity (-Ret)	Intensity (27°C)/ Intensity (37°C)	Rosetta ΔΔG (REU)	C_α Distance to Retinal Centroid (Å)	Biological Hydrophobicity ΔΔG (kcal/ mol)	9-cis-Retinal Sensitivity *	Temperature Sensitivity *
A 299 S	0.73	0.85	0.150	12.22	0.726	-	-
A 299 H	0.69	0.81	6.810	12.22	1.351	-	-
A 299 P	0.70	0.91	5.000	12.22	1.204	-	-
A 299 R	0.65	0.90	5.920	12.22	1.796	-	-
A 299 E	0.76	0.85	8.790	12.22	1.625	-	-
A 299 K	0.67	0.89	9.050	12.22	2.122	-	-
I 300 L	0.76	0.86	1.100	15.28	0.004	-	-
I 300 V	0.75	0.85	1.320	15.28	0.255	-	-
I 300 C	0.79	0.86	5.140	15.28	0.371	-	-
I 300 M	0.75	0.90	4.220	15.28	0.370	-	-
I 300 A	0.75	0.91	5.150	15.28	0.543	-	-
I 300 T	0.78	0.84	3.330	15.28	0.910	-	-
I 300 G	0.81	0.93	6.460	15.28	0.915	-	-
I 300 S	0.78	0.94	4.980	15.28	1.141	-	-
I 300 N	0.82	0.83	5.710	15.28	1.808	-	-
I 300 H	1.04	1.22	7.250	15.28	1.675	+	+
I 300 P	1.21	1.39	16.940	15.28	1.542	+	+
I 300 Q	1.04	1.21	4.070	15.28	1.805	+	+
I 300 R	0.93	0.95	5.820	15.28	1.894	-	-
I 300 K	0.92	0.95	8.040	15.28	2.292	-	-
Y 301 L	0.76	0.91	-2.800	15.70	-0.659	-	-
Y 301 F	0.71	0.81	-0.490	15.70	-0.551	-	-
Y 301 V	0.79	0.83	-1.770	15.70	-0.435	-	-
Y 301 C	0.75	0.85	2.160	15.70	-0.375	-	-
Y 301 M	0.78	0.90	-2.200	15.70	-0.376	-	-
Y 301 A	0.75	0.86	0.440	15.70	-0.269	-	-
Y 301 W	0.73	0.79	-0.570	15.70	-0.273	-	-
Y 301 T	0.78	0.85	0.880	15.70	-0.040	-	-
Y 301 Y	0.79	0.78	nan	15.70	0.000	-	-
Y 301 G	0.73	0.83	2.730	15.70	-0.004	-	-
Y 301 S	0.79	0.84	1.850	15.70	0.143	-	-
Y 301 P	0.73	0.85	16.580	15.70	0.400	-	-
Y 301 Q	0.69	0.82	0.070	15.70	0.565	-	-
Y 301 R	0.68	0.91	-2.360	15.70	0.488	-	-
Y 301 E	0.83	0.85	2.720	15.70	0.642	-	-
Y 301 K	0.68	0.80	-0.990	15.70	0.827	-	-
Y 301 D	0.75	0.79	3.210	15.70	0.833	-	-
N 302 L	0.73	0.85	2.510	16.29	-1.655	-	-
N 302 V	0.70	0.83	6.080	16.29	-1.327	-	-
N 302 C	0.76	0.81	7.600	16.29	-1.277	-	-
N 302 A	0.68	0.90	10.670	16.29	-1.159	-	-
N 302 T	0.71	0.77	6.990	16.29	-0.894	-	-

Mutation	Intensity (+Ret)/ Intensity (-Ret)	Intensity (27°C)/ Intensity (37°C)	Rosetta ΔΔG (REU)	C_α Distance to Retinal Centroid (Å)	Biological Hydrophobicity ΔΔG (kcal/ mol)	9-cis-Retinal Sensitivity *	Temperature Sensitivity *
N 302 Y	0.63	0.77	2.020	16.29	-0.892	-	-
N 302 G	0.67	0.88	0.280	16.29	-0.793	-	-
N 302 S	0.68	0.85	9.550	16.29	-0.599	-	-
N 302 N	0.68	0.82	nan	16.29	0.000	-	-
N 302 H	0.79	0.82	4.210	16.29	-0.108	-	-
N 302 Q	0.75	0.80	10.940	16.29	-0.025	-	-
N 302 R	0.70	0.85	8.200	16.29	-0.343	-	-
N 302 E	0.88	0.91	13.670	16.29	0.099	-	-
N 302 K	0.71	0.91	6.810	16.29	0.258	-	-
N 302 D	0.70	0.84	13.290	16.29	0.302	-	-
P 303 I	0.73	0.95	-6.870	19.35	-1.440	-	-
P 303 L	1.23	1.51	-5.700	19.35	-1.530	+	+
P 303 C	0.83	0.87	-4.200	19.35	-1.083	-	-
P 303 A	0.81	0.77	-5.170	19.35	-0.971	-	-
P 303 Y	1.05	1.73	-2.700	19.35	-0.818	+	+
P 303 G	0.75	0.88	-4.160	19.35	-0.553	-	-
P 303 S	0.80	0.93	-10.770	19.35	-0.354	-	-
P 303 P	0.67	0.92	nan	19.35	0.000	-	-
P 303 R	0.75	0.88	-8.860	19.35	-0.317	-	-
V 304 I	0.72	0.86	-1.000	20.69	-0.213	-	-
V 304 L	0.78	0.85	-2.540	20.69	-0.303	-	-
V 304 F	0.70	0.73	-0.740	20.69	-0.198	-	-
V 304 V	0.77	0.86	nan	20.69	0.000	-	-
V 304 C	0.72	0.83	1.750	20.69	0.011	-	-
V 304 M	0.79	0.91	-0.110	20.69	0.011	-	-
V 304 A	0.72	0.84	1.420	20.69	0.074	-	-
V 304 W	0.82	0.65	-0.370	20.69	-0.207	-	-
V 304 T	0.73	0.85	0.510	20.69	0.205	-	-
V 304 G	0.74	0.92	4.410	20.69	0.362	-	-
V 304 S	0.81	0.78	1.740	20.69	0.486	-	-
V 304 N	1.25	0.69	2.950	20.69	0.893	+	-
V 304 P	0.70	0.82	13.610	20.69	0.711	-	-
V 304 R	1.00	0.93	2.260	20.69	0.372	+	-
V 304 E	1.17	0.98	5.730	20.69	0.967	+	-
V 304 D	0.92	0.90	5.200	20.69	0.986	-	-
I 305 I	0.77	0.87	nan	20.60	0.000	-	-
I 305 L	0.75	0.90	4.320	20.60	-0.126	-	-
I 305 F	0.73	0.87	3.950	20.60	-0.018	-	-
I 305 V	0.73	0.87	5.120	20.60	0.206	-	-
I 305 C	0.76	0.88	4.760	20.60	0.211	-	-
I 305 A	0.77	0.93	5.360	20.60	0.266	-	-
I 305 W	0.83	0.97	5.360	20.60	-0.112	-	-

Mutation	Intensity (+Ret)/ Intensity (-Ret)	Intensity (27°C)/ Intensity (37°C)	Rosetta ΔΔG (REU)	C_α Distance to Retinal Centroid (Å)	Biological Hydrophobicity ΔΔG (kcal/ mol)	9-cis-Retinal Sensitivity *	Temperature Sensitivity *
I 305 T	0.77	0.85	6.160	20.60	0.373	-	-
I 305 Y	1.18	1.26	5.420	20.60	0.144	+	+
I 305 S	0.86	0.99	5.650	20.60	0.701	-	-
I 305 N	1.28	0.97	6.970	20.60	1.114	+	-
I 305 P	0.70	0.97	16.790	20.60	0.920	-	-
I 305 R	1.00	0.92	5.010	20.60	0.465	+	-
I 305 E	1.29	1.32	6.990	20.60	1.192	+	+
I 305 K	0.97	0.88	7.260	20.60	1.021	+	-
I 305 D	1.05	0.90	7.440	20.60	1.138	+	-
Y 306 I	0.68	1.03	-0.880	22.21	-0.039	-	-
Y 306 L	0.71	0.90	-3.730	22.21	-0.264	-	-
Y 306 F	0.70	0.89	0.920	22.21	-0.112	-	-
Y 306 V	0.67	0.91	-0.450	22.21	0.214	-	-
Y 306 C	0.70	0.82	0.830	22.21	0.217	-	-
Y 306 A	0.72	0.85	0.440	22.21	0.280	-	-
Y 306 W	0.78	0.94	0.230	22.21	-0.307	-	-
Y 306 T	0.71	0.86	-1.320	22.21	0.384	-	-
Y 306 Y	0.74	0.86	nan	22.21	0.000	-	-
Y 306 G	0.72	0.87	2.010	22.21	0.709	-	-
Y 306 S	0.74	0.85	0.250	22.21	0.844	-	-
Y 306 N	0.78	0.92	2.620	22.21	1.327	-	-
Y 306 H	0.72	0.83	3.100	22.21	1.277	-	-
Y 306 P	0.69	0.87	7.330	22.21	1.093	-	-
Y 306 Q	0.72	0.88	1.560	22.21	1.240	-	-
Y 306 R	0.68	0.93	-1.110	22.21	0.433	-	-
Y 306 E	0.84	0.87	2.200	22.21	1.420	-	-
Y 306 K	1.01	0.95	1.330	22.21	1.102	+	-
I 307 I	0.77	0.87	nan	24.91	0.000	-	-
I 307 L	0.71	0.80	0.390	24.91	-0.216	-	-
I 307 F	0.74	0.86	-0.240	24.91	-0.098	-	-
I 307 V	0.72	0.93	0.880	24.91	0.179	-	-
I 307 C	0.68	0.90	4.340	24.91	0.179	-	-
I 307 A	0.69	0.86	2.080	24.91	0.221	-	-
I 307 W	0.39	0.78	-0.740	24.91	-0.242	-	-
I 307 T	0.76	0.83	1.970	24.91	0.281	-	-
I 307 Y	0.79	0.96	-1.090	24.91	-0.040	-	-
I 307 G	0.75	0.91	1.980	24.91	0.585	-	-
I 307 S	0.74	0.87	2.410	24.91	0.682	-	-
I 307 P	0.82	0.89	11.010	24.91	0.868	-	-
I 307 Q	0.93	0.73	1.670	24.91	0.976	-	-
I 307 R	1.06	1.24	1.100	24.91	0.285	+	+
I 307 E	1.30	0.98	4.200	24.91	1.146	+	-

Mutation	Intensity (+Ret)/ Intensity (-Ret)	Intensity (27°C)/ Intensity (37°C)	Rosetta ΔΔG (REU)	C_α Distance to Retinal Centroid (Å)	Biological Hydrophobicity ΔΔG (kcal/ mol)	9-cis-Retinal Sensitivity *	Temperature Sensitivity *
I 307 D	1.11	0.92	4.460	24.91	0.945	+	-
M 308 I	0.73	0.86	1.340	25.68	-0.098	-	-
M 308 L	0.78	0.84	-3.430	25.68	-0.266	-	-
M 308 F	0.75	0.82	0.670	25.68	-0.188	-	-
M 308 V	0.73	0.88	1.170	25.68	0.000	-	-
M 308 T	0.67	0.86	-0.110	25.68	0.047	-	-
M 308 Y	0.76	0.72	0.750	25.68	-0.130	-	-
M 308 G	0.71	0.85	2.630	25.68	0.255	-	-
M 308 S	0.73	0.86	0.670	25.68	0.304	-	-
M 308 P	0.93	0.85	13.870	25.68	0.402	-	-
M 308 R	0.80	0.87	-0.330	25.68	0.038	-	-
M 309 I	0.72	0.89	-0.640	25.92	-0.109	-	-
M 309 L	0.76	0.86	-0.170	25.92	-0.359	-	-
M 309 F	0.66	0.82	-0.940	25.92	-0.260	-	-
M 309 V	0.74	0.85	0.140	25.92	0.000	-	-
M 309 C	0.74	0.87	2.990	25.92	0.000	-	-
M 309 A	0.76	0.79	0.700	25.92	0.023	-	-
M 309 W	0.72	0.80	1.580	25.92	-0.245	-	-
M 309 T	0.73	0.81	-2.370	25.92	0.044	-	-
M 309 Y	0.83	0.74	0.680	25.92	-0.113	-	-
M 309 G	0.77	0.84	-0.040	25.92	0.346	-	-
M 309 S	0.72	0.82	1.390	25.92	0.396	-	-
M 309 N	0.83	0.76	3.610	25.92	0.672	-	-
M 309 H	0.73	0.77	-0.060	25.92	0.671	-	-
M 309 Q	0.76	0.94	1.000	25.92	0.574	-	-
M 309 R	0.70	0.88	0.220	25.92	0.027	-	-
M 309 E	0.73	0.92	2.320	25.92	0.739	-	-
M 309 K	0.77	0.84	0.080	25.92	0.332	-	-
M 309 D	0.81	0.99	3.670	25.92	0.470	-	-

***** Variants with intensity ratios greater than one standard deviation from the mean were classified as sensitive (+) to 9-*cis*-retinal or temperature, otherwise they were classified as insensitive (-).
